# Acute transcriptional up-regulation specific to osteoblasts/osteoclasts in medaka fish immediately after exposure to microgravity

**DOI:** 10.1038/srep39545

**Published:** 2016-12-22

**Authors:** Masahiro Chatani, Hiroya Morimoto, Kazuhiro Takeyama, Akiko Mantoku, Naoki Tanigawa, Koji Kubota, Hiromi Suzuki, Satoko Uchida, Fumiaki Tanigaki, Masaki Shirakawa, Oleg Gusev, Vladimir Sychev, Yoshiro Takano, Takehiko Itoh, Akira Kudo

**Affiliations:** 1 Graduate School of Bioscience and Biotechnology, Tokyo Institute of Technology, Yokohama 226-8501, Japan; 2 Chiyoda Corporation, Yokohama 220-8765, Japan; 3 Department of Science and Applications, Japan Space Forum, Tokyo 101-0062, Japan; 4 Japan Aerospace Exploration Agency, Tsukuba 305-8505, Japan; 5 Institute of Fundamental Medicine and Biology, Kazan Federal University, Kazan 420008, Russia; 6 SSC RF-Institute of Biomedical Problems RAS, Moscow, Russia; 7 Section of Biostructural Science, Graduate School of Medical and Dental Sciences, Tokyo Medical and Dental University, Tokyo 113-8549, Japan

## Abstract

Bone loss is a serious problem in spaceflight; however, the initial action of microgravity has not been identified. To examine this action, we performed live-imaging of animals during a space mission followed by transcriptome analysis using medaka transgenic lines expressing osteoblast and osteoclast-specific promoter-driven GFP and DsRed. In live-imaging for osteoblasts, the intensity of *osterix*- or *osteocalcin*-DsRed fluorescence in pharyngeal bones was significantly enhanced 1 day after launch; and this enhancement continued for 8 or 5 days. In osteoclasts, the signals of *TRAP*-GFP and *MMP9*-DsRed were highly increased at days 4 and 6 after launch in flight. HiSeq from pharyngeal bones of juvenile fish at day 2 after launch showed up-regulation of 2 osteoblast- and 3 osteoclast- related genes. Gene ontology analysis for the whole-body showed that transcription of genes in the category “nucleus” was significantly enhanced; particularly, transcription-regulators were more up-regulated at day 2 than at day 6. Lastly, we identified 5 genes, *c-fos*, *jun-B-like*, *pai-1*, *ddit4* and *tsc22d3*, which were up-regulated commonly in the whole-body at days 2 and 6, and in the pharyngeal bone at day 2. Our results suggested that exposure to microgravity immediately induced dynamic alteration of gene expression levels in osteoblasts and osteoclasts.

In the animal body, various cellular stimuli such as heat shock^1^, oxidative^2^, and hypoxic^3^ stresses have been studied attentively. In spaceflight, when gravitational alteration occurs rapidly with the shift to microgravity, changes in fluid shift and blood pressure quickly take place in the human body^4^, leading to hemodynamic adaptation^5^ and alteration of vasoreactivity, accompanied by up-regulation of the NO/cGMP pathway, as was found in an *in vitro* study^6^. These responses to the extreme change in gravity continuously happen at the whole-body level; however, the molecular mechanisms of such responses to “microgravitational stress” remain unclear.

Bone loss in astronauts, which is one of severe health problems, is observed in a spaceflight lasting for a few months, which loss is reminiscent of that for senile osteoporosis on the ground. It is recognized that understanding the potential action of this microgravity environment toward bone loss should contribute to progress in the fields related to the effect of mechanical stress on bone, as well as to clinical application for osteoporosis. To investigate the mechanism of bone loss during spaceflight, it is important to study the initial response immediately after the initial exposure to microgravity, because this response represents the trigger for bone loss. As to symptoms that appear early in orbit, the loss of calcium starts at least 10 days after launch^7^,^8^; and the assessment of bone quality revealed a loss of bone in short-duration spaceflight for 20 days^9^. These reports suggest that osteoblasts and osteoclasts undergo changes immediately after launch. In fact, *in vitro* experiments conducted during a short-term parabolic flight showed changes in gene expression and cellular cytoskeleton in human chondrocytes^10^,^11^. However, the nature of the initial response *in vivo* to microgravity in bone tissues is unclear.

One way to answer this question is to perform animal experiments at the ISS (International Space Station). In a previous study, we found that in skeletogenesis of the vertebral body and pharyngeal bone in medaka, their osteoblasts and osteoclasts revealed properties similar to those of their mammalian counterparts^12^,^13^,^14^,^15^. In osteoblast differentiation, osterix is a typical marker of early osteoblasts; and osteocalcin, one of late osteoblasts^16^, whereas TRAP (tartrate-resistant acid phosphatase), cathepsin K, and MMP9 are markers of osteoclasts^17^. In medaka as in mammals these cells are differentiated from TRAP, cathepsin K, and MMP9-positive mononuclear cells into multinuclear osteoclasts^13^,^14^,^15^. Furthermore, the *c-fms* (the receptor of M-CSF)-deficient zebrafish shows a reduced number of osteoclasts, resulting in a bone modeling defect^14^, which finding indicates the essential function of M-CSF and *c-fms* in fish as well as in mammals^18^. Moreover, RANKL, the essential osteoclast differentiation factor expressed in osteoblasts, is common between mammals and medaka fish^19^. All evidence taken together indicates that the basic molecular mechanism underlying the differentiation of osteoclasts is common between mammals and fish and that the interaction of these cells with osteoblasts plays a crucial role in osteoclast differentiation in medaka as well. To identify osteoblasts and osteoclasts *in-vivo*, we previously developed a medaka *osterix* promoter-DsRed transgenic line for the visualization of osteoblasts^20^ and a medaka *TRAP* promoter-GFP transgenic line for that of osteoclasts^14^. Finally, we established a double transgenic line of *osterix*-DsRed and *TRAP*-GFP^21^ to examine the cooperation between osteoblasts and osteoclasts in the same animal. In the analysis of pharyngeal bones, by using this double transgenic line we showed the important role of osteoblasts for controlling osteoclasts to modify the attachment bone during tooth replacement in medaka pharyngeal teeth^15^. The row of attachment bones is resorbed at the anterior side where most developed functional teeth are located, and generated at the posterior side where teeth are newly erupting, which actions cause continuous tooth replacement. Osteoclasts and osteoblasts are located at attachment bones separately, with mature osteoclasts being localized at the resorbing side and osteoblasts gathered at the generating side.

When medaka fish at 6 weeks after hatching were launched to the ISS in 2012 and reared there for 2 months, activation of osteoclasts together with bone loss occurred in the flight fish^21^. In that study, to examine the alteration of gene expression early in orbit, we preserved specimens with RNAlater at days 2 and 6 after launch. Another way to study growing tissues under microgravity in space is to perform experiments using three-dimensional cultures^22^; however, such experiments have not yet been performed at the ISS. In our present study, to examine the early effects of microgravity on bone cells, we embedded transgenic medaka larvae in a gel for a live-imaging study in space in 2014, and observed signals by fluorescence microscopy at the ISS via remote operation from Tsukuba Space Center. For this experiment, we utilized 4 different double medaka transgenic lines and, in particular, investigated up-regulation of fluorescent signals of osteoblasts and osteoclasts in these double transgenic lines as an important way to study osteoblast-osteoclast interaction under microgravity.

In addition, we examined the pattern of gene expression in these transgenic fish by transcriptome analysis. HiSeq analysis of the pharyngeal bones showed the enhanced expression of osteoblast- and osteoclast-related genes. Furthermore, GO (gene ontology) analysis showed the up-regulation of AP-1-, GR- and TGF-β-related genes that coincided with osteoclast activation. Our results about live-imaging and transcriptome analysis may prompt the establishment of a new field in gravitational biology.

## Results

### Enhancement of osteoblast signals under microgravity

**Figure 1:**
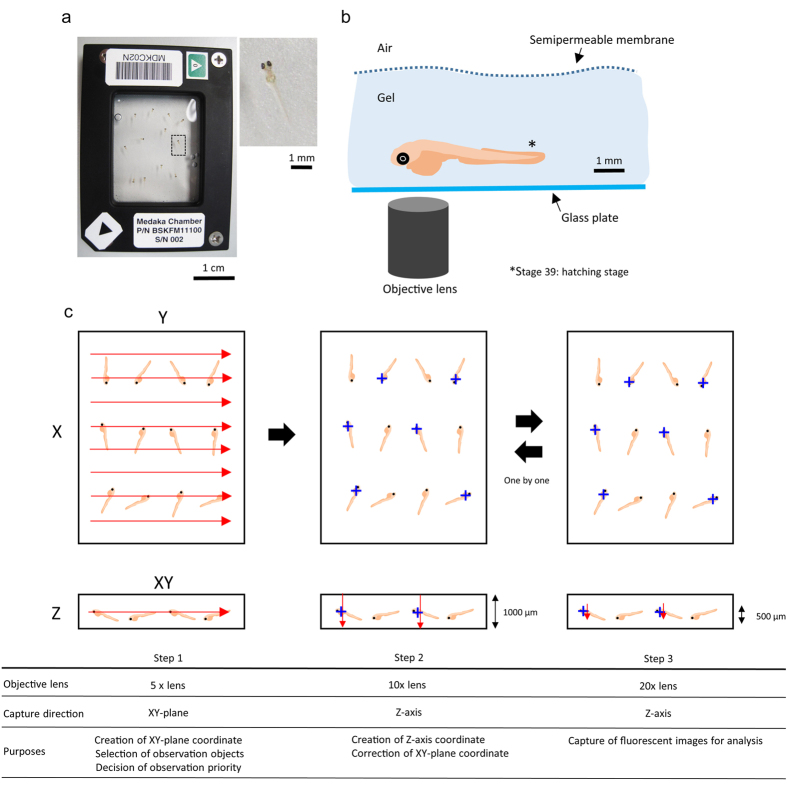
Live-imaging system at the ISS. (**a**) Left photo shows a top view of the medaka chamber. Scale bar = 1 cm. Right photo shows a whole image of a medaka larva that is seen in the enlarged view of the black-squared area in the medaka chamber. Scale bar = 1 mm. (**b**) Cartoon showing a lateral view of a medaka larva embedded in the Mebiol gel. The fish at stage 39 were observed via fluorescence microscopy from the bottom side. Scale bar = 1 mm. (**c**) Order of observation via remote operation. Three steps were required to get accurate location of medaka larvae. Step 1: At first, XY coordinate was created by using a 5x objective lens. The red arrows show the direction of capture on the XY plane. The observed objects were selected. Step 2: XY coordinates were corrected, and the Z coordinate was created by using the 10x objective lens. The blue crosses show the observed objects whose ventral side was oriented toward the glass plate for observation via an objective lens; and red arrows, the direction of capturing Z-axis with a width of 1000 μm. Step 3: Clear fluorescent images were captured by using a 20x objective lens with a width of 500 μm. The steps are summarized in the table at the bottom of Fig. 1.

**Figure 2:**
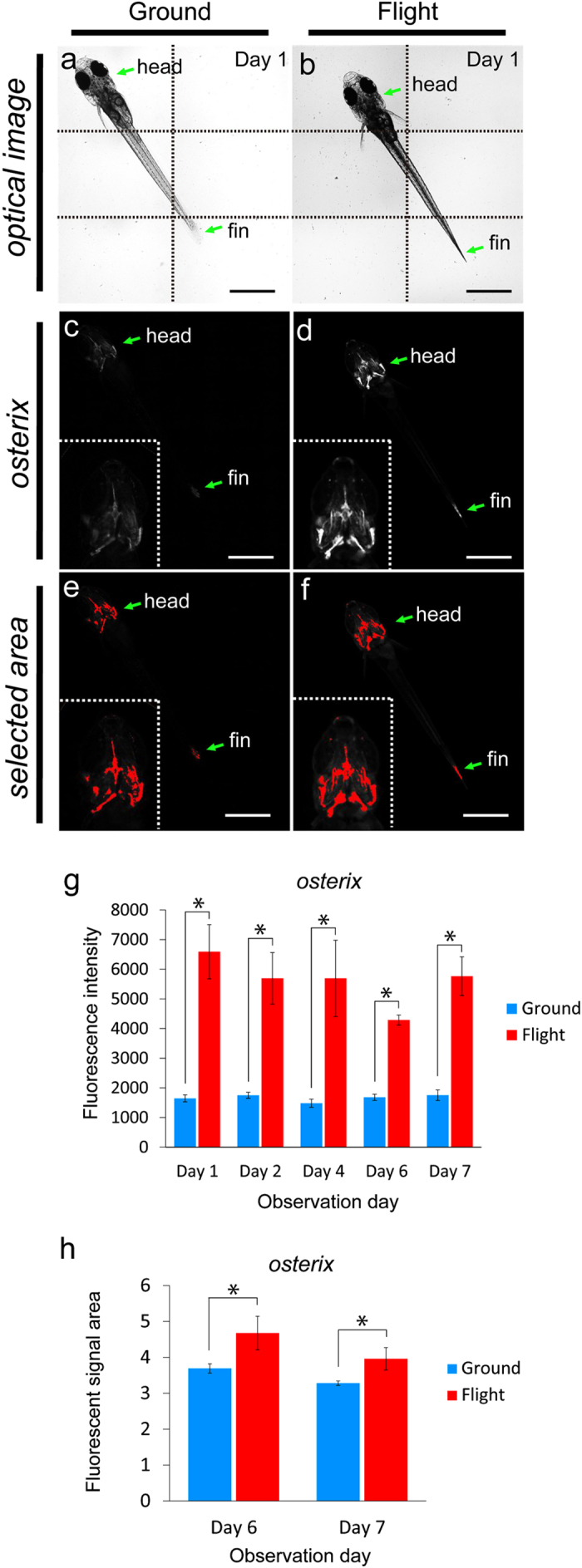
Whole-body imaging of the *osterix*-DsRed transgenic line. (**a**–**f**) The left-side images show the same ground control at day 1; and the right-side images, the same flight medaka at day 1. Arrows point to the head and fin region. All images show ventral views. Scale bars = 1 mm. (**a**,**b**) Montage images were made from 6 captured optical images, divided by dotted lines. (**c**,**d**) The white region shows an *osterix*-DsRed fluorescent signal. Embedded views show the enlarged head region. The images were captured under the same conditions between flight and ground samples shown in Supplementary Table S2. (**e**,**f**) The red region shows the area selected for measurement of the intensity of fluorescent signals by using ImageJ software. Embedded views show the enlarged head region. (**g**) The fluorescent intensity from day 1 to day 7 of observation day constantly increased in the flight group. Ground, n = 5 (all days). Flight, n = 3 (day 1), 3 (day 2), 4 (day 4), 3 (day 6), and 3 (day 7), as shown in Supplementary Table S1. *P < 0.05, Student’s *t*-test. Error bars, s.e.m. (**h**) The fluorescent signal area was increased in the flight group compared with that in the ground group at days 6 and 7. Ground, n = 5 (day 6, 7); flight, n = 3 (day 6, 7), as shown in “Supplementary Table S1.” *P < 0.05, Student’s *t*-test. Error bars, s.e.m.

**Figure 3:**
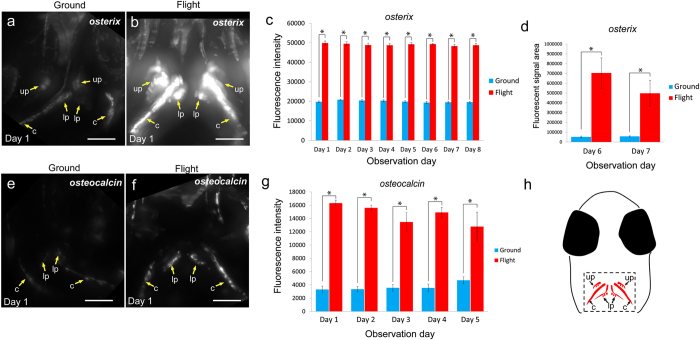
Live imaging for osteoblasts. Evaluation of *osterix*-DsRed (**a–d**) and *osteocalcin*-DsRed (**e–h**) fluorescent signals. (**a**,**b,e,f**) Stacked ventral images in the pharyngeal region at day 1 for the ground (**a,e**) and flight (**b,f**) groups. The images were captured under the same conditions between flight and ground samples shown in Supplementary Table S2 and constructed by Z-projection done with ImageJ software. Yellow arrows show bones with osteoblasts localized on them. up: upper pharyngeal bone, lp: lower pharyngeal bone, c: cleithrum. Scale bars = 50 μm. (**c**,**g**) The fluorescence intensity of *osterix* (**c**) and *osteocalcin* (**g**) constantly increased during the flight. The numbers of *osterix* line fish: ground, n = 9 (day 1), 10 (day 2), 10 (day 3), 10 (day 4), 11 (day 5), 10 (day 6), 11 (day 7), and 11 (day 8); flight, n = 6 (day 1), 6 (day 2), 6 (day 3), 7 (day 4), 7 (day 5), 6 (day 6), 5 (day 7), and 4 (day 8). Numbers of *osteocalcin* line fish: ground, n = 6 (day 1), 7 (day 2), 7 (day 3), 6 (day 4), and 3 (day 5); flight, n = 3 (day 1), 3 (day 2), 5 (day 3), 4 (day 4), and 4 (day 5). These numbers are shown in Supplementary Table S1. *P < 0.05, Student’s *t*-test. Error bars, s.e.m. (**d**) The *osterix*-positive area was significantly increased in the flight group at days 6 and 7. *P < 0.05, Student’s *t*-test. Error bars, s.e.m. (**h**) Schematic diagram of the ventral view of a medaka larva head. The dotted rectangle shows the observation area in Fig. 3a,b,e,f and Fig. 4a,b,d,e. up: upper pharyngeal bone, lp: lower pharyngeal bone, c: cleithrum.

To find alteration of signal intensity and area of osteoblasts and osteoclasts, we observed the fluorescent signals in living medaka for 8 days at the ISS as shown in Methods and Table 1. Twelve larvae at stage 39 were embedded in “medaka chambers” (Fig. 1a), in which live larvae in a gel were covered with a semipermeable membrane. Pharyngeal bones, around which many osteoblasts and osteoclasts were localized, were clearly observed from the ventral side (Supplementary Fig. S1). This ventral side was oriented toward the glass plate for observation via an objective lens (Fig. 1b). For capturing images by use of a 20x lens, we carried out observation in 3 steps, because the posture of the living larvae was constantly changing (Fig. 1c). The experimental schedule for live-imaging of the 4 double transgenic lines, *osterix*-DsRed/*TRAP*-GFP, *osteocalcin*-DsRed/*TRAP*-GFP, *MMP9*-DsRed/*RANKL*-GFP, and *cox2*-GFP/*TRAP*-DsRed during 8 days in flight and on the ground is shown in Supplementary Table S1. To perform the imaging analyses for both the flight and ground samples under the same conditions, we manipulated both fluorescence microscopes, one at ISS and the other on the ground at the same gain value and the same appropriate exposure time (Supplementary Table S2). Firstly, the capture images of DsRed in the *osterix*-DsRed/*TRAP*-GFP line, which fluorescence visualizes the early stage of osteoblasts, revealed high expression in the flight group (Fig. 2a–f). To examine the effect of microgravity on the whole-body, we observed overall this transgenic medaka, and found that the intensity of the fluorescent signals was enhanced in the whole-body (Fig. 2g,h). Then, we focused on the pharyngeal bone region, in which bone turnover is high and sensitive to microgravity^21^, and observed ground and flight samples at high magnification with a 20x lens (Fig. 3a,b) to examine the details of fluorescent signals in osteoblasts. When the fluorescent signal was measured in the pharyngeal bone region including the cleithrum (Fig. 3h), the results revealed that in the flight group, this intensity was increased compared with that in the ground group over the 8-day observation period (Fig. 3c). The signal-positive area was also increased about 8.0 fold or more (Fig. 3d). Next, to examine the late stage of osteoblasts, we measured the intensity and area of DsRed signals in the *osteocalcin*-DsRed/*TRAP*-GFP line and found a large increase in intensity in the flight group (Fig. 3e–h); however, no statistically significant increase in area was found (data not shown). To remove any possibility of a contribution of hypergravity exerted during the launch into space to the level of fluorescent signals, we performed a hypergravity experiment, and the results showed no significant alteration of signals (Supplementary Fig. S2). This result suggested that the hypergravity occurring at launch seems to have had no detectable effect on the *osterix*-DsRed signals.

### Increase in the fluorescence intensity in osteoclasts

**Figure 4:**
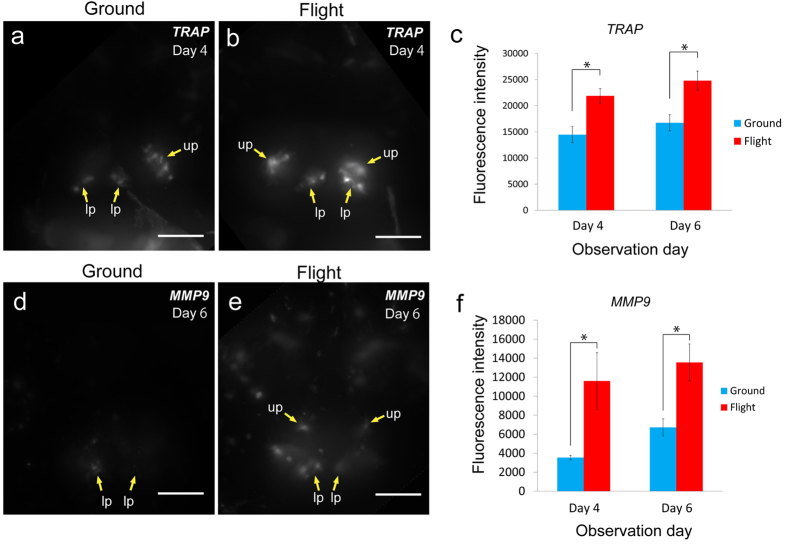
Live imaging for osteoclasts. Evaluation of *TRAP*-GFP (**a–c**) and *MMP9*-DsRed (**d**–**f**) fluorescent signals. (**a**,**b**,**d**,**e**) Stacked ventral images in the pharyngeal region at day 4 (*TRAP*-GFP) and at day 6 (*MMP9*-DsRed) for the ground (**a,d**) and flight (**b,e**) groups. The images were captured under the same conditions between flight and ground samples shown in Supplementary Table S2 and constructed by Z-projection by use of ImageJ software. Yellow arrows point to bones with osteoclasts localized on them. up: upper pharyngeal bone, lp: lower pharyngeal bone. Scale bars = 50 μm. (**c**,**f**) The fluorescence intensity for *TRAP* (**d**) and for *MMP9* (**f**) increased during the flight at days 4 and 6 of the observation period. Numbers of *TRAP* line fish: ground, n = 14 (day 4), 16 (day 6); flight, n = 9 (day 4), 7 (day 6). Numbers of *MMP9* line fish: ground, n = 6 (day 4), 6 (day 6); flight, n = 4 (day 4), 3 (day 6). These numbers are shown in Supplementary Table S1. *P < 0.05, Student’s *t*-test. Error bars, s.e.m.

In osteoclast development, since the larva at stage 39 shows the initial phase for osteoclastogenesis in the pharyngeal bone region, we observed fluorescent signals of larvae at this stage in *TRAP*-GFP or in *MMP9*-DsRed fish. MMP9 as well as TRAP^14^ is a typical marker of osteoclasts in medaka (Supplementary Fig. S3). Our results showed that the intensity of the *TRAP*-GFP-positive signal was increased at days 4 and 6 after launch in the flight group compared with that in the ground group (Fig. 4a–c), whereas the signal area was not significantly altered (data not shown). The intensity of *MMP9*-DsRed signals was also increased at days 4 and 6 after launch in the flight group (Fig. 4d–f), though there was no significant alteration of the area (data not shown). Thus the fluorescent signals driven by osteoblast- and osteoclast- specific promoters were enhanced in the flight group. Both *RANKL*-GFP, which replicates the expression pattern of endogenous RANKL (data not shown), which is the key factor for osteoclast differentiation^23^, and *cox2*-GFP^24^ (data not shown) lines showed no significant difference in the fluorescence intensity between the ground and flight groups (Supplementary Fig. S4).

### Co-localization of osteoblasts and osteoclasts under microgravity

**Figure 5:**
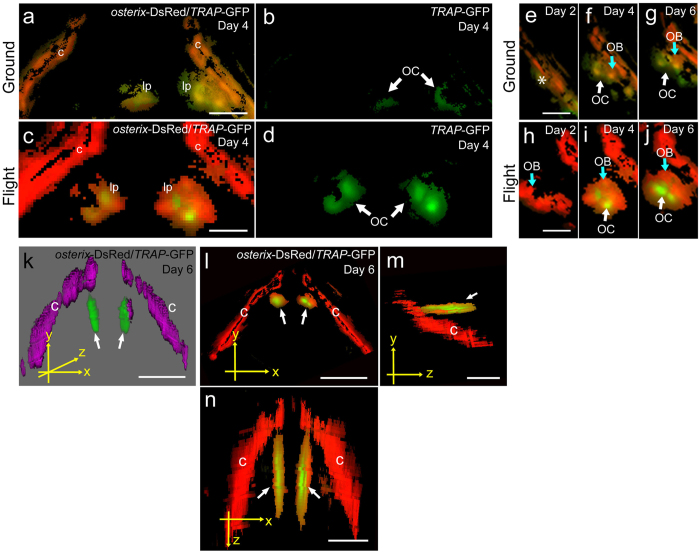
3D imaging for osteoblasts and osteoclasts in flight group. (**a–n**) The merged images were captured by 3D views for *osterix*-DsRed and *TRAP*-GFP in the pharyngeal bone region of the double transgenic line. The images were processed by use of Fiji software. (**a**,**c**) The pharyngeal bone region in the ground control (**a**) and the flight (**c**) group at day 4. lp, lower pharyngeal bone; c, cleithrum. Scale bars = 20 μm. (**b**,**d**) Images for *TRAP*-GFP in the pharyngeal bone region of “a” and “c”, respectively. GFP signals identify osteoclasts (OC). (**e–j**) Development of osteoblasts (OB) and osteoclasts (OC) in the lower pharyngeal bone region in the ground control (**e–g**) and flight (**h–j**) groups. Scale bars = 20 μm. No signals of *osterix*-DsRed and *TRAP*-GFP were detected in the pharyngeal bone region (e, asterisk). The *osterix*-DsRed and *TRAP*-GFP were weakly expressed at the low pharyngeal bone region at days 4 and 6 in the ground control (**f,g**). *The osterix*-DsRed in osteoblasts (OB) was highly expressed at the low pharyngeal bone region at day 2 (**h**). *TRAP*-GFP signals in osteoclasts (OC) appeared near *osterix*-DsRed signals at day 4 (**i**). *TRAP*-GFP signals were spread at the low pharyngeal bone region at day 6 (**j**). (**k**–**n**) Localization of osteoblasts and osteoclasts. White arrows show GFP signals in the lower pharyngeal bone. c, cleithrum. 3D image for *osterix*-DsRed/*TRAP*-GFP at day 6 (**k**). Osteoblasts and osteoclasts are shown in magenta and green, respectively. This image was not analyzed by the deconvolution algorithm, and thus the signals appear as an out-of-focus blur. The views were captured on the x-y plane (**l**), y-z plane (**m**), and x-z plane (**n**) from 3D images. Scale bars = 50 μm.

The interaction of osteoblasts and osteoclasts is important for bone remodeling. It has been reported that there are many osteoblasts and osteoclasts in pharyngeal bones^21^, and osteoblasts are important for osteoclastogenesis in these bones^15^. To study the co-localization of osteoblasts and osteoclasts, we observed *osterix*-DsRed/*TRAP*-GFP merged images (Fig. 5). DsRed signals for osteoblasts were enhanced in the flight group, and the GFP signals for osteoclasts localized near osteoblasts were also enhanced during flight (Fig. 5a–d). The *TRAP*-GFP signals emerged at the lower pharyngeal bone region; and compared with the intensity of the ground control they increased in intensity near the osteoblasts highly expressing *osterix* in the flight medaka (Fig. 5e–j). Furthermore, we confirmed the localization of GFP and DsRed signals in the pharyngeal bone region in flight medaka by performing three-dimensional (3D) analysis (Fig. 5k–n). Figure 5k shows a 3D image of the distribution of the fluorescent proteins. The pharyngeal bone was localized at the inside of the cleithrum, as shown in Supplementary Fig. S1.

### Alteration of bone-related gene expression in the pharyngeal bone region by HiSeq

The results of live-imaging showed the enhancement of fluorescent signals in both osteoblasts and osteoclasts under microgravity. To examine alteration of the gene expression levels in bone tissues, we extracted RNAs from the pharyngeal bone region in medaka juvenile at day 2 after launch. Unfortunately, because the amount of RNAs from small pharyngeal bones was extremely low, the RNAs of individual fish were mixed to perform HiSeq analysis of the flight versus ground group. Our results showed that 2 osteoblast-related genes, *col10a1* and *osteocalcin*, and 3 osteoclast-related genes, *acp5* (*TRAP*), *cathepsin K*, and *MMP9*, were significantly up-regulated in the flight group (Table 2). Regarding *osterix* mRNA, the expression level showed 7.99 fold increase in the flight, though this data showed less statistically importance (data not shown).

### GO analysis for gene expression of whole-body medaka in flight by RNA-Seq

**Figure 6:**
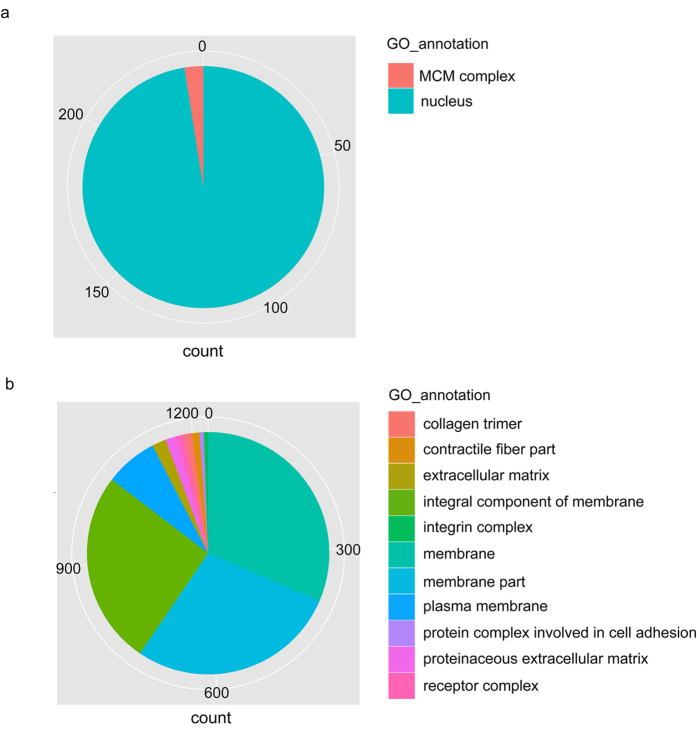
Annotations for up- and down-regulated genes in flight-group medaka by GO analysis. (**a**) The pie chart shows the number of up-regulated genes, which were assigned by GO; *Nucleus*, 258: *MCM complex*, 6. (**b**) Pie chart showing the number of down-regulated genes assigned by GO: *Collagen trimer*, 14; *contractile fiber part*, 12; *extracellular matrix*, 24; *integral component of membrane*, 333; *integrin complex,* 8; *membrane*, 408; membrane part, 365; plasma membrane, 93; protein complex involved in cell adhesion, 8; proteinaceous extracellular matrix, 19; receptor complex, 14. The results in “a” and “b” show “*cellular component*” in GO domains.

To examine the alteration of gene expression levels early in orbit, we focused on the common up- and down-regulated genes in the whole-body at days 2 and 6 after launch. RNA-Seq analysis was performed on total RNAs extracted from the whole-body medaka. We examined over 20,130 (90.3%) and 21,062 (94.5%) expressed genes at days 2 and 6, respectively. With a false-discovery rate (FDR) of <=0.2, 2,958 up-regulated (S > G) genes (13.3% of all genes) and 3,631 down-regulated (S < G) genes (16.3% of all genes) were shown to be differentially expressed genes (DEG) by the RNA-Seq analysis. The number of common genes for up- and down-regulation between days 2 and 6 was 105 and 48, respectively, and categorized according to the following classification: Molecular function, Biological process, and Cellular component (Supplementary Table S3). Figure 6 shows the ratio of enriched GO annotations for up- and down-regulated genes in the “*cellular component*” in the GO domain. In particular, the GO analysis showed that GO:0005634 (nucleus) was significantly enhanced in up-regulated genes under the microgravity, whereas GO:0005886 (plasma membrane) was significantly enhanced in down-regulated genes.

### Up-regulation of the molecules acting in the nucleus

GO analysis showed up-regulation of the molecules that act in the nucleus. Of the 105 genes that were up-regulated in the whole-body medaka at days 2 and 6, 33 genes were specifically found as molecules that act in the nucleus. Thirty-one of these 33 genes were up-regulated at day 2 compared with their expression at day 6, suggesting that enhancement of transcription occurred immediately after exposure to microgravity (Supplementary Table S4). In addition, the gene expression levels of MCM (minichromosome maintenance) family genes, mcm7, mcm5, mcm4, and mcm3 were significantly increased at day 6 (Supplementary Table S5).

### Alteration of the gene expression levels of cell-surface molecules

Forty-eight genes were down-regulated in the whole-body medaka at days 2 and 6. Nineteen genes and 29 genes were down-regulated extremely at day 2 and 6, respectively. For the highly down-regulated genes for membrane proteins in the flight group, we classified them into 4 categories of membrane proteins: cell adhesion proteins (7 genes), channel and transporter proteins (3 genes), receptor proteins (2 genes), and membrane-related proteins (12 genes), as shown in Supplementary Table S6. Especially, the solute carrier family and the claudin family were characterized in both up- and down-regulated gene expression profiles: *slc13a5* and *slc25138A* were up-regulated, *slc12a3* was down-regulated, *claudin-5* was up-regulated, and *claudin-7* and *claudin-like protein ZF-A89-like* were down-regulated (Supplementary Table S6). These findings suggest that the expression levels of *slcs* and *claudins* were influenced by gravity.

### Up-regulation of molecules in pharyngeal bone tissues and whole-body

To identify the common up-regulated genes related to bone tissues among 105 genes that were up-regulated in the whole-body at days 2 and 6, we examined the overlapped genes in the transcriptome between the pharyngeal bone at day 2 and the whole-body at days 2 and 6. Our results showed that only 5 genes, i.e., *jun-B-like* and *c-fos* for the AP-1 family, *pai-1* (*Plasminogen activator inhibitor type 1*), *ddit4* (*DNA damage-inducible transcript 4 protein*), and *tsc22d3* (*TSC 22 domain family member 3*), were found to be significantly up-regulated genes (Table 3).

## Discussion

Under microgravity, there are several changes in the animal body, such as fluid shift, increase in blood pressure, and dizziness. In particular, bone mineral density is decreased under microgravity^25^; but it is unclear how osteoblasts or osteoclasts respond early in orbit. In spaceflight, there are 2 phases in the adaptation to a specified environment under microgravity; one is the initial extreme alteration from ground 1 G to μG early in orbit, and the other is the adaptation to chronic stress during long-term flight. It is important to understand the initial response; however, previous studies were performed either *in vitro* or by using artificial microgravity. In our present study, we took 2 approaches, i.e., live imaging and transcriptome analysis, to examine the initial response to microgravity. Especially, the live-imaging analysis is useful to examine real-time alterations of cells under microgravity. We observed living animals (medaka) at the ISS for the first time via remote operation from Tsukuba Space Center, and found by live-imaging that the fluorescent signals of osteoblasts as well as those of osteoclasts were significantly increased over those for the ground control. In the whole-body fish, the *osterix*-DsRed and *osteocalcin*-DsRed signals in osteoblasts were increased in intensity, indicating that osteoblasts were directly affected by microgravity regardless of osteoclast commitment.

We propose the following possible mechanism for the up-regulation of *osterix*, *osteocalcin*, *TRAP*, and *MMP9* genes under microgravity: Osteoblasts and osteoclasts are located on bones, which are most sensitive to gravity alteration because they are the tissue with the highest density in the body. Bone-specific genes such as *osterix* and *osteocalcin* may have properties affording a response to gravity. Both *osterix* and *osteocalcin* transcription was up-regulated at the same time: day 1 as shown in the fluorescent intensity. This is different from normal osteoblast differentiation in which *osterix* and *osteocalcin* expressions appear at the early and the later stage, respectively, during osteoblast differentiation. At the ISS, this disorder of osteoblasts causes activation of osteoclasts, or osteoclasts may respond directly to the gravity alteration in a different manner, resulting in enhanced expression of *TRAP* and *MMP9* genes.

The transcriptome analysis of pharyngeal bones showed significant up-regulation of 2 osteoblast-related genes and 3 osteoclast-related genes in the flight group: *col10a1* and *osteocalcin* and for the former and *acp5 (TRAP)*, *cathepsin*, and *MMP9* for the latter. Enhanced gene expression of *osteocalcin*, *TRAP*, and *MMP9* was consistent with the results of the live-imaging study.

Gene ontology analysis using mRNAs from the whole-body fish at days 2 and 6 showed significant up-regulation of genes involved in “nucleus regulation”. Consistently, it has been reported that in parabolic flight experiments performed *in vitro*, unexpected effects on cells are observed, mainly on molecules related to transcription^10^. It is considered that cells adapt to a change (microgravity) by rearranging their transcriptional apparatus to counteract alterations in gene expressions as a reaction to a stimulus under microgravity. Among our findings, 2 of them were especially important. Firstly, of the genes involved in regulation of the nucleus, we found that 5 of the 23 nucleus-related genes selected in the whole-body transcriptome analysis were related to osteoblast or osteoclast function, and were up-regulated to a greater extent at day 2 than at day 6. Regarding these 5 genes, *klf9* and *klf2* are regulated in the macrophage network via the glucocorticoid receptor^26^, *foxa3* is a crucial regulator of hypertrophic chondrocyte differentiation^27^, *maf* promotes osteoblast differentiation^28^, and *CCAAT/enhancer-binding protein delta* is involved in inflammation^29^. Secondly, we found that the MCM (minichromosome maintenance complex) family, which is the DNA helicase complex required for DNA replication, was up-regulated at day 6. Especially, *mcm3* in osteoblasts was reported to be up-regulated by irradiation^30^. Taken together, our results demonstrate that cells immediately produce the necessary transcription factors and remodel the chromatin structure in their nucleus. Interestingly, 2 transcription factor genes, *gadd45* and *klf2*, were reportedly up-regulated under hypergravity in the another fish model, zebrafish^31^; and these 2 genes were also up-regulated under microgravity (Supplementary Table S4). Furthermore, *c-fos*, *jun-B-like*, and *jun dimerization protein 2-like* were also up-regulated under microgravity (Supplementary Table S4); and in a clinorotation study, which is commonly used to compensate for the unilateral effect of gravity, FOS-JUN transcription factors were regulated by the GADD45B factor for their global expression^32^. Taken together, these findings indicate that there may exist a general mechanism against the gravity.

In the analysis of commonly up-regulated genes in the whole-body fish at days 2 and 6, and in pharyngeal bones at day 2, we found that 5 of them, i.e., *c-fos*, *jun-B-like*, *pai-1*, *ddit4,* and *tsc22d3*, were commonly up-regulated. c-Fos and jun-b are immediate early-response genes that include response to alteration of gravity^33^,^34^; and among the members of the jun family, junB is quickly activated, as was shown in a clinostat experiment^34^. This jun member is a part of the inducible transcription factor complex AP-1, which is a positive regulator for controlling the formation of primary osteoblasts and osteoclasts^35^. Furthermore, TGF-β induces pai-1^36^ and tsc22^37^,^38^. Omata *et al*. reported that c-fos and Smads can activate osteoclasts, in which Smads are involved in the TGF-β signaling pathway^39^, a pathway potentially activated by mechanical stress.

Previous studies showed that glucocorticoid induces pai-1^37^, ddit4^40^, and tsc22d3^41^ and that the transcription factor AP1 regulates glucocorticoid receptor (GR) binding at the chromatin level^42^. GRs are possibly involved in osteoclast activation, because GR and AP-1 synergistically activate transcription, in which AP-1 guides GR binding. Pai-1 and Tsc22d3 are activated by the combination of TGF-β and GR^26^,^36^,^41^,^43^,^44^, whereas ddit4 is enhanced by GR^40^. During spaceflight, blood pressure changes early in orbit^45^,^46^. It is also known that the stress of elevated blood pressure produces nitric oxide (NO)^47^ or NO reduces blood pressure^48^. Recently, it was shown that GR and NO have a synergistic function^49^; and interestingly, it was reported that stimulation by NO induces *tsc22d3*, *ddit4* and *klf2*^50^, and that preeclamptic plasma down-regulates *klf9*, *tsc22d3*, *cebpb*, *ddit4* and *jdp2*^51^. In our results, expressions of these genes were altered under microgravity, which thus suggests that NO-GR signaling is related to the “microgravitational stress”. Taken together, available data indicate that the GR is the main molecule involved in the effect of microgravity on cells, consistent with our observations of activation of GRs in medaka fish reared long-term in the ISS^21^. Finally, to clarify the GR action under microgravity, further analyses are required. We plan to further investigate the function of these genes in our next space experiment.

## Methods

### Double-transgenic medaka lines

We established 4 medaka double-transgenic lines: *osterix*-DsRed^20^/*TRAP*-GFP^14^,^21^, *osteocalcin*-DsRed^12^/*TRAP*-GFP^14^,^21^, *RANKL*-GFP/*MMP9*-DsRed, and *cox2*-GFP^24^/*TRAP*-DsRed^24^.

*MMP9*-DsRed line: A 3.2-kb upstream regulatory region of the medaka mmp9 gene was amplified by using the following primers: 5′-GCGAAGCTTCATGTTGCTAAGTCTCAGAGTC-3′ (forward) and 5′-GCGAATTCTTTGAGATCTAATGTGGACTAGTG-3′ (reverse). The amplified fragments were cloned into the TA cloning vector. We then digested the 5′-half fragment with HindIII and EcoRI, and subcloned it into the HindIII/EcoRI site of the I-Sce1 backbone vector, which was subsequently inserted into DsRed-Express/SV40polyA (Clontech) at the HindIII/AflII sites in the pBSKI2 vector (AMAGEN).

*RANKL*-GFP line: The fosmid clone golwfno356_d02 including the *rankl* genomic locus was obtained from an ensemble database and NBRP Medaka (https://shigen.nig.ac.jp/medaka/), utilized for homologous recombination with an EGFP-kanamycin cassette by using the protocol previously described^52^. The EGFP cassette was amplified by using the following primers: 5′-AAGCCTCCAGAGTCCGGGAGCGTGGGGCCGATCCGCACGCGACGCGCGTCTCCACCG GTCGCCAC CATGG-3′ (forward) and 5′-GCGCTCGACAGGCTCCTCGGAGCGCGCGCGCGCGCACAGGCTGCCCTTACGAGGCTATGGCAGGGCCTGC-3′ (reverse). The underlines show the homologous recombination sites. The PCR products were recombined into exon 1 of the medaka *rankl* gene.

### Two space experiments for study of the initial response to microgravity

We performed space experiments twice using medaka fish for the study of the early response to microgravity (Table 1). In 2012, 6 juvenile fish each were preserved by RNAlater at the ISS at days 2 and 6 after launch. In 2014, the fluorescent signals from 24 larvae fish of 4 double transgenic lines were observed at the ISS for *in vivo* imaging under microgravity.

The experiments were performed in accordance with policies and protocols approved by the Japan Aerospace Exploration Agency (JAXA) Institutional Animal Care and Use Committee. In the experiment at the ISS in 2012, at days 2 and 6 after launch, medaka fish were transported into the “Fish Fixation Apparatus” for analysis of mRNA expression levels. The buffer used in the Fish Fixation Apparatus was RNAlater (Sigma-Aldrich, MO, USA). Specimens were frozen and stored at −95 °C for later mRNA analyses. These specimens were maintained at −95 °C and transported from NASA to Japan.

In the next experiment, medaka larvae were placed in the “Medaka Chamber” and flown on the Soyuz flight TMA-10M (Roscosmos, Russia) in 2014. After arrival at KIBO, which is the space laboratory of Japan in the International Space Station (ISS), the medaka chambers were set under a fluorescence microscope for live imaging for 8 days.

### Embedding of medaka larvae for *in vivo* imaging

Cab, an inbred wild-type strain of the medaka (O. latipes), was used throughout this study. Fish were kept under a photoperiod of 14 h light/10 h darkness at 28 °C. Eggs were obtained and kept at 25–28 °C after collection. After hatching at day 9, larvae were embedded in Mebiol Gel (Mebiol Inc). Mebiol Gel has a unique property; i.e., it is a liquid at a low temperature, but turns into a gel upon being warmed up.

For preparation of the gel solution, 0.5 ppm methylene blue in water including 1% streptomycin and 0.57% penicillin was filtered, after which 12 ml of the solution was added to the Mebiol Gel container. For dissolution, the containers were shaken for 2 days at 4 °C.

Hatching larvae were incubated on ice for 10 minutes for anesthesia, and 12 larvae were lined up on the glass plate of a chamber. Promptly, the chamber was filled with 2.1 ml of gel solution, after which its upper side was covered with a semipermeable membrane. As shown in Supplementary Fig. S1, pharyngeal bones were clearly observed from the ventral side. In this experiment, the ventral side was oriented toward the glass plate for observation via an objective lens (Fig. 1b).

Medaka chamber (#1) contained *osterix*-DsRed/*TRAP*-GFP (n = 10) and *RANKL*-GFP/*MMP9*-DsRed (n = 2); and Medaka chamber (#2), *RANKL*-GFP/*MMP9*-DsRed (n = 3), *osteocalcin*-DsRed/*TRAP*-GFP (n = 5), and *cox2*-GFP/*TRAP*-DsRed (n = 4). Three chambers including backup samples were prepared for #1 and #2, respectively.

The experiment for the ground control was carried out under the same conditions as used for the flight group.

### Microscope Observation System

The Microscope Observation System consisted of a microscope, a power supply, and the control unit containing a VGA-NTSC converter and an Experiment Laptop Terminal (ELT). This system was used to conduct fluorescence observations and also supported live imaging of medaka transgenic lines. We manipulated this system under the same measurement conditions both at ISS and on the ground. During the experiment at the ISS, the crew set up the medaka sample at the ISS, and remote observations and operations were conducted by command from Earth via the ELT (Experiment Laptop Terminal). The recorded image files were transferred back to Earth. The microscope used was an inverted vertical illumination fluorescence microscope (DMI6000B, Leica Microsystems) partially modified to fit the space environment. All microscope operations on the stage, the object lens revolver, the fluorescence filter turret, and the capacitor were controlled electrically^53^.

### Live imaging of medaka fish

Twelve larvae at stage 39 were embedded in one “Medaka Chamber” (Fig. 1a), where larvae in Mebiol gel (Mebiol Inc.) were covered with a gas-permeable membrane to remain alive. The temperature for this experiment was maintained between 19–23 °C. The chambers including fish larvae were shaded all day. The numbers of fish in the flight and ground groups are shown in Supplementary Table S1. The growth of fish in the flight group was similar to that of those in the ground group, and the larvae in both conditions were at stage 39 when they were embedded in the gel.

The microscope used was a modified fluorescence microscope (DMI 6000B, Leica Microscope Systems Co.), which was controlled by a specialized software. This software was able to receive a command from the ground control and execute the microscope’s actions continuously by using a batch process. Fluorescent signals were observed with the filter set and the LED light source optimized for each signal. Collection of images of live larvae in random spatial distribution was performed in 3 steps. In the first step, the XY coordinates of each larva was set approximately by using a 5x objective lens. In the second step, these coordinates were narrowed down; and the Z coordinate was set within the limits of 1000 μm by using a 10x objective lens. The interval of all continuous images was 100 μm in the head region. In the final step, the tack-sharp Z-stack images within the limits of 500 μm were captured by using a 20x lens. The interval of all continuous images was 5 μm in the pharyngeal bone region. These steps were summarized in “Fig. 1”.

In the ground control group, images were captured under the same conditions as used for the flight group, and the temperature was controlled at around 21 °C. We measured fluorescent signals using the same time course on the ground and in the flight. We confirmed that there was no significant difference in the calibration of the microscope by using the background luminance caused by the excitation light for both GFP and DsRed fluorescence. We checked this calibration value by using *cox-2-GFP* signals at the head of the transgenic line, which showed the constitutive signals and found a similar level of signals in both ISS and the ground (data not shown).

### Quantitative analysis of fluorescent signals

The intensity and area of GFP and DsRed signals were calculated by use of ImageJ software (ImageJ). The fluorescent signal area was selected by the process *Image*/*Adjust*/*Threshold* in ImageJ. Adjustment of the parameter in “*Threshold*” shows the adequate selected area as a “red region” on the window of ImageJ. Next, the intensity and the area were calculated by the process *Analyze*/*Measure* (“*Limit to threshold*” is selected in “*Set Measurements*”). The fluorescent signal area was cropped by “*Polygon sections”* when necessary. The captured images during flight were noised as small spots by cosmic rays. These small spots were canceled by removal of outliers (radius: 2.0 pixels, threshold: 50, and bright outliers). The range of each observation was adjusted enough to contain the fluorescence of tissues. The observation of pharyngeal bones in the flight samples covered 101 slices, and that of the ground control, about 55 slices, by focus adjustment with manual operation for the minimum range. The images were stacked in a single file. As described above, we selected the fluorescent signal area and calculated it with the process, *Analyze*/*Histogram,* by the measurement of both “area” and “intensity” for all images, which was shown as a stacked file.

### Statistics of fluorescent signals

The fluorescent signal data are shown as the mean ± s.e.m. The statistical analysis was performed by the one-way analysis of variance, followed by unpaired Student’s *t*-test. A P-value < 0.05 was conventionally considered statistically significant.

### Analysis for the localization of osteoblasts and osteoclasts

For *osterix*-DsRed and *TRAP*-GFP in the pharyngeal bone region, the three-dimensional images of the fluorescent proteins were constructed by using Fiji, an image processing package.

### Hypergravity experiment

To confirm the effect of hypergravity at launch, we reproduced the hypergravity condition^54^. The embedded *osterix*-DsRed transgenic medaka fish in a gel were continuously exposed to 4.3 G for 2 minutes, 2.7 G for 2 minutes 40 seconds, and 3.75 G for 4 minutes 5 seconds. The fluorescent signals for *osterix*-DsRed were observed before 1 day (day-1), immediately after (day 0), 1 day after (day 1) and 2 days after (day 2) exposure to the hypergravity by using a centrifuge customized for medaka fish (LIX-140SP, Tomy Seiko Co., Ltd). The larvae were kept at 25 °C for the experiment.

### RNA preparation

Medaka fish were preserved by use of RNAlater at days 2 and 6 after launch into spaceflight in 2012. For flight specimens, RNAs from pharyngeal bones, or whole bodies were extracted at day 2 (n = 4), or at days 2 (n = 3) and 6 (n = 3) after launch. Ground control fish were preserved at Baikonur at day 2 after launch, and RNAs from pharyngeal bones, or whole bodies were extracted as day 2 (n = 10) control, or days 2 (n = 3) and 6 (n = 3) control.

### Whole transcriptome analysis by HiSeq

Since a small amount of RNA was extracted from pharyngeal bones at day 2 after launch, flight- and ground- cDNAs were synthesized by using a mixture of RNAs of all extracted samples. The method was followed by the protocol previously reported^21^.

### GO (gene ontology) analysis of whole-body RNA

RNAs from whole bodies were examined by RNA-Seq analysis. The library was of the single-end type. Reads for each condition were mapped to the reference genome. FPKM (fragments per kilobase of exon per million mapped fragments) was calculated for each library, and the difference in FPKM between flight and control groups was compared with consideration of biological duplication. We identified DEGs (differentially expressed genes) by performing the *t*-test.

Three patterns, day 2 after launch (n = 3), day 6 after launch (n = 3), and both days 2 and 6 (n = 6), were compared with each other; and the replication number was indicated. In confirmation of common or non-common DEG, a candidate DEG was picked up; and then we performed the GO analysis.

### Statistics of transcriptome

In the GO analysis, the threshold of p-values in the analysis was set at 0.05. False-discovery rates (FDR) were calculated, and the threshold in the analysis was set at 0.2.

In the gene expression analysis, the threshold of p-values in the analysis was set at 0.05. False-discovery rates (FDR) were calculated and the threshold in the analysis was set at 0.3.

### cDNA sequence registration

We registered all RNA-Seq data at the DDBJ (the DNA Data Bank of Japan; http://www.ddbj.nig.ac.jp/). Raw reads of the transcriptome analysis in this study are deposited in the DDBJ Sequence Read Archive under BioProject ID: PRJDB5292.

## Additional Information

**How to cite this article:** Chatani, M. *et al*. Acute transcriptional up-regulation specific to osteoblasts/osteoclasts in medaka fish immediately after exposure to microgravity. *Sci. Rep.* **6**, 39545; doi: 10.1038/srep39545 (2016).

**Publisher's note:** Springer Nature remains neutral with regard to jurisdictional claims in published maps and institutional affiliations.

## Supplementary Material

Supplementary Figures and Tables

## Figures and Tables

**Table 1:** Time schedules for preparation of medaka in Baikonur and experiments in ISS.

Date (GMT)	Time course	Event
A part of Long-term experiment		
2012/10/23	18 hrs before launch	Preparation of fish at three weeks after hatch
2012/10/23	Launch at 10:51	Launch of Soyuz
2012/10/25	2 days after launch	Docking of Soyuz to ISS
2012/10/25	8 hrs after docking	The start of experiment in AQH
2012/10/25	10 hrs after docking	Fish were preserved by RNAlater (day 2)
2012/10/29	6 days after launch	Fish were preserved by RNAlater (day 6)
Short-term experiment		
2014/1/26	9 days before launch	Egg collecting
2014/2/4	32-25 hrs before launch	Preparation of observation chambers for hatched fish
2014/2/5	Launch at 16:23	Launch of Progress
2014/2/5	6 hrs after launch	Docking of Progress to ISS
2014/2/6	21 hrs after docking	Preparation of experiment
2014/2/7	36 hrs after launch	Observation day 1 (Start of live imaging)
2014/2/8	3 days after launch	Observation day 2
2014/2/9	4 days after launch	Observation day 3
2014/2/10	5 days after launch	Observation day 4
2014/2/11	6 days after launch	Observation day 5
2014/2/12	7 days after launch	Observation day 6
2014/2/13	8 days after launch	Observation day 7
2014/2/14	9 days after launch	Observation day 8 (Finish of live imaging)

GMT: Greenwich Mean Time.

**Table 2:** Genes involved in bone matrix homeostasis significantly modulated in flight (F) versus ground (G) in pharyngeal bone tissue at day 2.

Gene ID	Gene title	Fold change (F/G)	p-value	q-value
***Osteoblast**-related gene*				
LOC100529176	col10a1	11.36	0.0066	0.21
LOC100529177	osteocalcin	16.48	0.0029	0.099
***Osteoclast**-related gene*				
LOC101175134	acp5 (TRAP)	209.56	1.78E-07	1.30E-05
LOC101164922	cathepsin K	60.07	6.39E-06	0.00033
LOC100125420	MMP9	20.31	0.00077	0.027

**Table 3:** The up-regulated genes in pharyngeal bone at day 2, which were commonly up-regulated in whole-body medaka at days 2 and 6 in flight (F) versus ground (G).

Gene ID	Gene title	FC (Day 2, F/G)	p-value	q-value
LOC100529196	plasminogen activator inhibitor type 1	21.03	0.0088	0.28
LOC101160111	DNA damage-inducible transcript 4 protein-like	16.05	0.0023	0.08
LOC101164062	transcription factor jun-B-like	17.35	0.0049	0.16
LOC100820712	c-fos	19.15	0.0016	0.05
LOC101161414	TSC22 domain family, member 3	12.17	0.0090	0.28

^*^FC means fold change.

## References

[b1] Sorger P. K. Heat shock factor and the heat shock response. Cell 65, 363–366 (1991). 2018972 10.1016/0092-8674(91)90452-5

[b2] Keyse S. M. & Emslie E. A. Oxidative stress and heat shock induce a human gene encoding a protein-tyrosine phosphatase. Nature 359, 644–7 (1992). 1406996 10.1038/359644a0

[b3] Rius J., Guma M., Schachtrup C., Akassoglou K., Zinkernagel A. S. . NF-κB links innate immunity to the hypoxic response through transcriptional regulation of HIF-1α. Nature 453, 807–811 (2008). 18432192 10.1038/nature06905 PMC2669289

[b4] Clement G. Fundamentals of Space Medicine 2nd edn (Kindle edition) Vol. 23 Space Technology Library (eds Werts James R. .) ch. 4.3.2, location 3923-3966 of 8799 (Springer, 2011).

[b5] Liu J., Verheyden B., Beckers F. & Aubert A. E. Haemodynamic adaptation during sudden gravity transitions. Eur. J. Appl. Physiol. 112, 79–89 (2012). 21479958 10.1007/s00421-011-1956-6

[b6] White A. R., Ryoo S., Bugaj L., Attarzadeh D. O., Thiyagarajan S. . Early changes in vasoreactivity after simulated microgravity are due to an upregulation of the endothelium-dependent nitric oxide/cGMP pathway. Eur. J. Appl. Physiol. 110, 395–404 (2010). 20512503 10.1007/s00421-010-1514-7

[b7] Rambaut P. C. & Johnston R. S. Prolonged weightlessness and calcium loss in man. Acta Astronaut. 6, 1113–1122 (1979). 11883480 10.1016/0094-5765(79)90059-6

[b8] Clement G. Fundamentals of Space Medicine 2nd edn (Kindle edition), Vol. 23 Space Technology Library (eds Werts James R. .) ch. 5.4.1, location 4726–4768 of 8799 (Springer, 2011).

[b9] McCarthy I., Goodship A., Herzog R., Oganov V., Stussi E. . Investigation of bone changes in microgravity during long and short duration space flight: Comparison of techniques. Eur. J. Clin. Invest. 30, 1044–1054 (2000). 11122319 10.1046/j.1365-2362.2000.00719.x

[b10] Wehland M., Aleshcheva G., Schulz H., Saar K., Hübner N. . Differential gene expression of human chondrocytes cultured under short-term altered gravity conditions during parabolic flight maneuvers. Cell Commun. Signal. 13, 1–13 (2015). 25889719 10.1186/s12964-015-0095-9 PMC4369370

[b11] Aleshcheva G., Wehland M., Sahana J., Bauer J., Corydon T. J. . Moderate alterations of the cytoskeleton in human chondrocytes after short-term microgravity produced by parabolic flight maneuvers could be prevented by up-regulation of BMP-2 and SOX-9. FASEB J. 29, 2303–2314 (2015). 25681461 10.1096/fj.14-268151

[b12] Inohaya K., Takano Y. & Kudo A. The teleost intervertebral region acts as a growth center of the centrum: *in vivo* visualization of osteoblasts and their progenitors in transgenic fish. Dev. Dyn. 236, 3031–3046 (2007). 17907202 10.1002/dvdy.21329

[b13] Nemoto Y., Higuchi K., Baba O., Kudo A. & Takano Y. Multinucleate osteoclasts in medaka as evidence of active bone remodeling. Bone. 40, 399–408 (2007). 17049327 10.1016/j.bone.2006.08.019

[b14] Chatani M., Takano Y. & Kudo A. Osteoclasts in bone modeling, as revealed by *in vivo* imaging, are essential for organogenesis in fish. Dev. Biol. 360, 96–109 (2011). 21963458 10.1016/j.ydbio.2011.09.013

[b15] Mantoku A., Chatani M., Aono K., Inohaya K. & Kudo A. Osteoblast and osteoclast behaviors in the turnover of attachment bones during medaka tooth replacement. Dev. Biol. 409, 370–381 (2016). 26658319 10.1016/j.ydbio.2015.12.002

[b16] Aubin J. E. Mesenchymal stem cells and osteoblast differentiation. Principles of Bone Biology third edition: 85–107, edited by Bilezikian .Academic Press (2008).

[b17] Takeshita S., Kaji K. & Kudo A. Identification and characterization of the new osteoclast progenitor with macrophage phenotypes being able to differentiate into mature osteoclasts. J. Bone Miner. Res. 15, 1477–88 (2000). 10934646 10.1359/jbmr.2000.15.8.1477

[b18] Dai X. M., Ryan G. R., Hapel A. J., Dominguez M. G., Russell R. G. . Targeted disruption of the mouse colony-stimulating factor 1 receptor gene results in osteopetrosis, mononuclear phagocyte deficiency, increased primitive progenitor cell frequencies, and reproductive defects. Blood 99, 111–120 (2002). 11756160 10.1182/blood.v99.1.111

[b19] To T. T., Witten P. E., Renn J., Bhattacharya D., Huysseune A. . Rankl-induced osteoclastogenesis leads to loss of mineralization in a medaka osteoporosis model. Development 139, 141–50 (2012). 22096076 10.1242/dev.071035

[b20] Inohaya K., Takano Y. & Kudo A. Production of Wnt4b by floor plate cells is essential for the segmental patterning of the vertebral column in medaka. Development 137, 1807–1813 (2010). 20460365 10.1242/dev.051540

[b21] Chatani M., Mantoku A., Takeyama K., Abduweli D., Sugamori Y. . Microgravity promotes osteoclast activity in medaka fish reared at the international space station. Sci. Rep. 5, 1–13 (2015). 10.1038/srep14172 PMC4585676 26387549

[b22] Unsworth B. R. & Lelkes P. I. Growing tissues in microgravity. Nat. Med. 4, 901–907 (1998). 9701241 10.1038/nm0898-901

[b23] Yasuda H., Shima N., Nakagawa N., Yamaguchi K., Kinosaki M. . Osteoclast differentiation factor is a ligand for osteoprotegerin/osteoclastogenesis-inhibitory factor and is identical to TRANCE/RANKL. Proc. Natl. Acad. Sci. USA 95, 3597–3602 (1998). 9520411 10.1073/pnas.95.7.3597 PMC19881

[b24] Takeyama K., Chatani M., Takano Y. & Kudo A. *In-vivo* imaging of the fracture healing in medaka revealed two types of osteoclasts before and after the callus formation by osteoblasts. Dev. Biol. 394, 292–304 (2014). 25131195 10.1016/j.ydbio.2014.08.007

[b25] Vico L., Collet P., Guignandon A., Lafage-Proust M.-H., Thomas T. . Effects of long-term microgravity exposure on cancellous and cortical weight-bearing bones of cosmonauts. Lancet 355, 1607–1611 (2000). 10821365 10.1016/s0140-6736(00)02217-0

[b26] Chinenov Y., Coppo M., Gupte R., Sacta M. a. & Rogatsky I. Glucocorticoid receptor coordinates transcription factor-dominated regulatory network in macrophages. BMC Genomics 15, 656 (2014). 25099603 10.1186/1471-2164-15-656 PMC4133603

[b27] Lonescu A., Kozhemyakina E., Nicolae C., Kaestner K. H., Olsen B. R. . FoxA family members are crucial regulators of the hypertrophic chondrocyte differentiation program. Dev. Cell 22, 927–939 (2012). 22595668 10.1016/j.devcel.2012.03.011 PMC3356573

[b28] Nishikawa K., Nakashima T., Takeda S., Isogai M., Hamada M. . Maf promotes osteoblast differentiation in mice by mediating the age-related switch in mesenchymal cell differentiation. J. Clin. Invest. 120, 3455–65 (2010). 20877012 10.1172/JCI42528 PMC2947225

[b29] Ko C.-Y., Chang W.-C. & Wang J.-M. Biological roles of CCAAT/Enhancer-binding protein delta during inflammation. J. Biomed. Sci. 22, 6 (2015). 25591788 10.1186/s12929-014-0110-2 PMC4318212

[b30] Yamamoto M., Tamura K., Hiratsuka K. & Abiko Y. Stimulation of MCM3 gene expression in osteoblast by low level laser irradiation. Lasers Med. Sci. 16, 213–217 (2001). 11482820 10.1007/pl00011357

[b31] Aceto J., Nourizadeh-Lillabadi R., Maree R., Dardenne N., Jeanray N. . Zebrafish bone and general physiology are differently affected by hormones or changes in gravity. PLoS ONE 10, e0126928 (2015). 26061167 10.1371/journal.pone.0126928 PMC4465622

[b32] Aceto J., Nourizadeh-Lillabadi R., Bradamante S., Maier J. A., Alestrom P. . Effects of microgravity simulation on zebrafish transcriptomes and bone physiology- exposure stating at 5 days post fertilization. Npj Microgravity 2, 16010 (2016). 10.1038/npjmgrav.2016.10 PMC5515515 28725727

[b33] Pompeiano M., D’Ascanio P., Centini C., Pompeiano O. & Balaban E. Short-term (FOS) and long-term (FRA) protein expression in rat locus coeruleus neurons during the neurolab mission: Contribution of altered gravitational fields, stress, and other factors. Neuroscience 115, 111–123 (2002). 12401326 10.1016/s0306-4522(02)00402-5

[b34] Granet C., Vico A. G. L., Alexandre C. & Lafage-Proust M.-H. MAP and src kinases control the induction of AP-1 members in response to changes in mechanical environment in osteoblastic cells. Cell. Signal. 14, 679–88 (2002). 12020768 10.1016/s0898-6568(02)00008-6

[b35] Kenner L., Hoebertz A., Beil F. T., Keon N., Karreth F. . Mice lacking JunB are osteopenic due to cell-autonomous osteoblast and osteoclast defects. J. Cell Biol. 164, 613–623 (2004). 14769860 10.1083/jcb.200308155 PMC2171977

[b36] Pan X.-Y., Wang Y., Su J., Huang G.-X., Cao D.-M. . The mechanism and significance of synergistic induction of the expression of plasminogen activator inhibitor-1 by glucocorticoid and transforming growth factor beta in human ovarian cancer cells. Mol. Cell. Endocrinol. 407, 37–45 (2015). 25770462 10.1016/j.mce.2015.03.005

[b37] Shibanuma M., Kuroki T. & Nose K. Isolation of a gene encoding a putative leucine zipper structure that is induced by transforming growth factor beta 1 and other growth factors. J. Biol. Chem. 267, 10219–24 (1992). 1587811

[b38] Tse W. K. F., Jiang Y.-J. & Wong C. K. C. Zebrafish transforming growth factor-β-stimulated clone 22 domain 3 (TSC22D3) plays critical roles in Bmp-dependent dorsoventral patterning via two deubiquitylating enzymes, Usp15 and Otud4. Biochim. Biophys. Acta 1830, 4584–93 (2013). 23665588 10.1016/j.bbagen.2013.05.006

[b39] Omata Y., Yasui T., Hirose J., Izawa N., Imai Y. . Genome-wide comprehensive analysis reveals critical cooperation between Smad and c-Fos in RANKL-induced osteoclastogenesis. J. Bone Miner. Res. 30, 869–877 (2014). 10.1002/jbmr.2418 25431176

[b40] Wong S., Tan K., Carey K. T., Fukushima A., Tiganis T. . Glucocorticoids stimulate hepatic and renal catecholamine inactivation by direct rapid induction of the dopamine sulfotransferase Sult1d1. Endocrinology 151, 185–194 (2010). 19966186 10.1210/en.2009-0590

[b41] Suarez P. E., Rodriguez E. G., Soundararajan R., Mérillat A.-M., Stehle J.-C. . The glucocorticoid-induced leucine zipper (gilz/Tsc22d3-2) gene locus plays a crucial role in male fertility. Mol. Endocrinol. 26, 1000–13 (2012). 22556341 10.1210/me.2011-1249 PMC5416992

[b42] Biddie S. C., John S., Sabo P. J., Thurman R. E., Johnson T. A. . Transcription factor AP1 potentiates chromatin accessibility and glucocorticoid receptor binding. Mol. Cell 43, 145–155 (2011). 21726817 10.1016/j.molcel.2011.06.016 PMC3138120

[b43] Takahashi H., Ikematsu K., Tsuda R. & Nakasono I. Increase in dual specificity phosphatase 1, TGF-beta stimulated gene 22, domain family protein 3 and Luc7 homolog (S. cerevisiae)-like messenger RNA after mechanical asphyxiation in the mouse lung. Leg. Med. (Tokyo). 11, 181–5 (2009). 19364672 10.1016/j.legalmed.2009.03.008

[b44] Nakamura M., Kitaura J., Enomoto Y., Lu Y., Nishimura K. . Transforming growth factor-β-stimulated clone-22 is a negative-feedback regulator of Ras/Raf signaling: Implications for tumorigenesis. Cancer Sci. 103, 26–33 (2012). 21943131 10.1111/j.1349-7006.2011.02108.x PMC11164176

[b45] Buckey J. C. J. R., Gaffney F. A., Lane L. D., Levine B. D., Watenpaugh D. E. . Central venous pressure in space. J. Appl Physiol. 81, 19–25 (1996). 8828643 10.1152/jappl.1996.81.1.19

[b46] Foldager N., Andersen T. A. E., Jessen F. B., Ellegaard P., Stadeager C. . Central venous pressure in humans during microgravity. J. Appl Physiol. 81, 408–12 (1996). 8828692 10.1152/jappl.1996.81.1.408

[b47] Hermann M., Flammer A. & Lüscher T. F. Nitric oxide in hypertension. J. Clin. Hypertens. (Greenwich). 8, 17–29 (2006). 17170603 10.1111/j.1524-6175.2006.06032.x PMC8109558

[b48] Sofronova S. I., Borzykh A. A., Gaynullina D. K., Kuzmin I. V., Shvetsova A. A. . Endothelial nitric oxide weakens arterial contractile responses and reduces blood pressure during early postnatal development in rats. Nitric Oxide. 55–56, 1–9 (2016). 10.1016/j.niox.2016.02.005 26923819

[b49] Cohen O., Ish-Shalom E., Kfir-Erenfeld S., Herr I. & Yefenof E. Nitric oxide and glucocorticoids synergize in inducing apoptosis of CD4^+^8^+^ thymocytes: implications for ‘Death by Neglect’ and T-cell function. Int. Immunol. 24, 783–91 (2012). 22949567 10.1093/intimm/dxs083

[b50] Turpaev K., Glatigny A., Bignon J., Delacroix H. & Drapier J.-C. Variation in gene expression profiles of human monocytic U937 cells exposed to various fluxes of nitric oxide. Free Radic. Biol. Med. 48, 298–305 (2010). 19892011 10.1016/j.freeradbiomed.2009.10.054

[b51] Calicchio R., Buffat C., Mathieu J. R., Salem N. B., Mehats C. . Preeclamptic plasma induces transcription modifications involving the AP-1 transcriptional regulator JDP2 in endothelial cells. Am. J. Pathol. 183, 1993–2006 (2013). 24120378 10.1016/j.ajpath.2013.08.020

[b52] Nakamura S., Saito D. & Tanaka M. Generation of transgenic medaka using modified bacterial artificial chromosome. Dev. Growth Differ. 50, 415–419 (2008). 18422685 10.1111/j.1440-169X.2008.01027.x

[b53] Handbook for Use of Laboratory in Kibo. Data of access: 10/12/2015, 79–82 at http://iss.jaxa.jp/kibo/library/fact/data/pmhandbook_en.pdf.

[b54] Soyuz user’s manual issue 1. Data of access: 10/09/2015, 3–2 at http://www.arianespace.com/launch-services-soyuz/Soyuz_Users_Manual_CSG_June06.pdf.

