# Supplementary material for: Acute transcriptional up-regulation specific to osteoblasts/osteoclasts in medaka fish immediately after exposure to microgravity
Source: Sci Rep. 2016 Dec 22;6:39545. doi: 10.1038/srep39545 (PMC5177882; doi:10.1038/srep39545)
Supplement: Supplementary Figures and Tables [file srep39545-s1.pdf]

Acute transcriptional up-regulation specific to osteoblasts/osteoclasts in medaka fish immediately after exposure to microgravity

Masahiro Chatani,[1,†] Hiroya Morimoto,[1] Kazuhiro Takeyama,[1] Akiko Mantoku,[1] Naoki Tanigawa,[2] Koji Kubota,[2] Hiromi Suzuki,[3] Satoko Uchida,[3] Fumiaki Tanigaki,[4] Masaki Shirakawa,[4] Oleg Gusev,[5,††] Vladimir Sychev,[6] Yoshiro Takano,[7] Takehiko Itoh,[1] and Akira Kudo[1]

[1] Graduate School of Bioscience and Biotechnology, Tokyo Institute of Technology, Yokohama 226-8501, Japan

[2] Chiyoda Corporation, Yokohama 220-8765, Japan

[3] Department of Science and Applications, Japan Space Forum, Tokyo 101-0062, Japan

[4] Japan Aerospace Exploration Agency, Tsukuba 305-8505, Japan

[5] Institute of Fundamental Medicine and Biology, Kazan Federal University, Kazan 420008, Russia

[6] SSC RF-Institute of Biomedical Problems RAS, Moscow, Russia

[7] Section of Biostructural Science, Graduate School of Medical and Dental Sciences, Tokyo Medical and Dental University, Tokyo 113-8549, Japan

[†] Current address: Department of Pharmacology, School of Dentistry, Showa University, Tokyo 142-8555, Japan

[††] Current address: RIKEN Innovation Center, RIKEN, Yokohama 230-0045, Japan

Corresponding author:

Akira Kudo, PhD

Department of Biological Information, Tokyo Institute of Technology

4259-B-33 Nagatsuta, Midori-ku, Yokohama 226-8501, Japan
Tel: 81-45-924-5718, Fax: 81-45-924-5718, E-mail: akudo@bio.titech.ac.jp

Running title: Initial response of osteoblasts and osteoclasts to microgravity

Supplementary Figure S1. Alizarin Red staining.

(**a**-**e**) The larva, 5.0 mm in total length, was taken 3 days post hatching. Ventral view of pharyngeal bones in the head region is seen (a). Scale bar = 100 µm. Enlarged views are focused on the lower pharyngeal bone (b), upper pharyngeal bone (c) and cleithrum (d). Lateral view of the head region is shown (e). Yellow arrows indicate the pharyngeal bones. Up, upper pharyngeal bone; lp, lower pharyngeal bone; c, cleithrum.

Supplementary Figure S2. No effect of hypergravity on osterix-DsRed signals.

(**a**) The fluorescent signals for *osterix*-DsRed were observed 1 day before (day -1), immediately after (day 0), 1 day after (day 1), and 2 days after (day 2) hypergravity for mimicking the condition of loaded gravity during the launch of Soyuz. Left column shows the 1G control; and the right one, the hypergravity group. Scale bars = 100 µm. (**b**) Quantitative analysis of the intensity of *osterix*-DsRed, which revealed no significant difference between the 1G control and hypergravity group. n.s., no significant difference.

Supplementary Figure S3. *MMP9*-positive cells overlap with *TRAP*-positive cells.

(**a**) Fluorescent images from ventral view in the head region of larva 5.3 mm in total length at 3 days after hatching. White arrows show the pharyngeal bone region. Merge (left), *TRAP*-GFP (middle), *MMP9*-DsRed (right). p, pigment cell. Scale bar = 200 µm.

(**b**) TRAP staining and Von Kossa staining for MMP9-DsRed/TRAP-GFP transgenic line 9 days after hatching (7 mm total length). Black arrows indicate TRAP-positive osteoclasts. Scale bar = 200 µm. (**c**) Fluorescent image of the same section seen in "b." The green and red colors show the *TRAP*-GFP and *MMP9*-DsRed signals, respectively. The white arrows point to the *TRAP*- and *MMP9*-positive cells. (**d-f**) Enlarged views of the yellow-dotted square area in "c." The merged image (d) shows the co-localization of *MMP9*-DsRed (e) and *TRAP*-GFP (f) in an osteoclast (white arrows). Scale bar = 20 µm.

Supplementary Figure S4. No significant difference in fluorescent signals in *RANKL*-GFP line between ground and flight medaka.

(**a**) Ventral view of RANKL-GFP transgenic line at day 1 after fertilization. *RANKL* was expressed at the pharyngeal bone region (yellow arrows in the dotted rectangle), and the fluorescent signal of RANKL was enhanced. Scale bar = 200 µm. (**b**) Fluorescent images of *RANKL*-GFP in the pharyngeal bone region of the ground (left) and flight (right) medaka in a gel, which images were captured by the automatic handling technique shown in Figure 1. The images were captured under the same conditions between flight and ground samples shown in Supplementary Table S2. Yellow arrows point to the lower pharyngeal bone region. Scale bars = 50 µm. (**c**) Comparison of the intensity of *RANKL*-GFP signals. Values are shown as mean ± s.e.m. Ground, n=6 (days 3, 4, 5); flight, n=3 (days 3, 4, 5), as given in Supplementary Table 1. n.s.; no significance by Student's *t*-test.

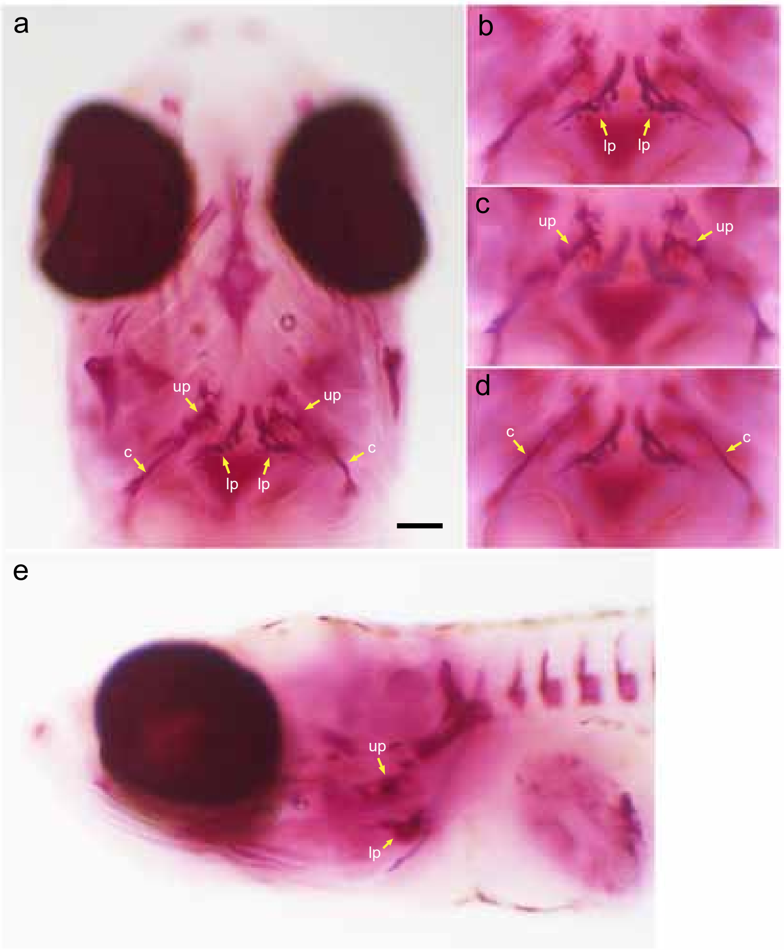

Supplementary Figure S1

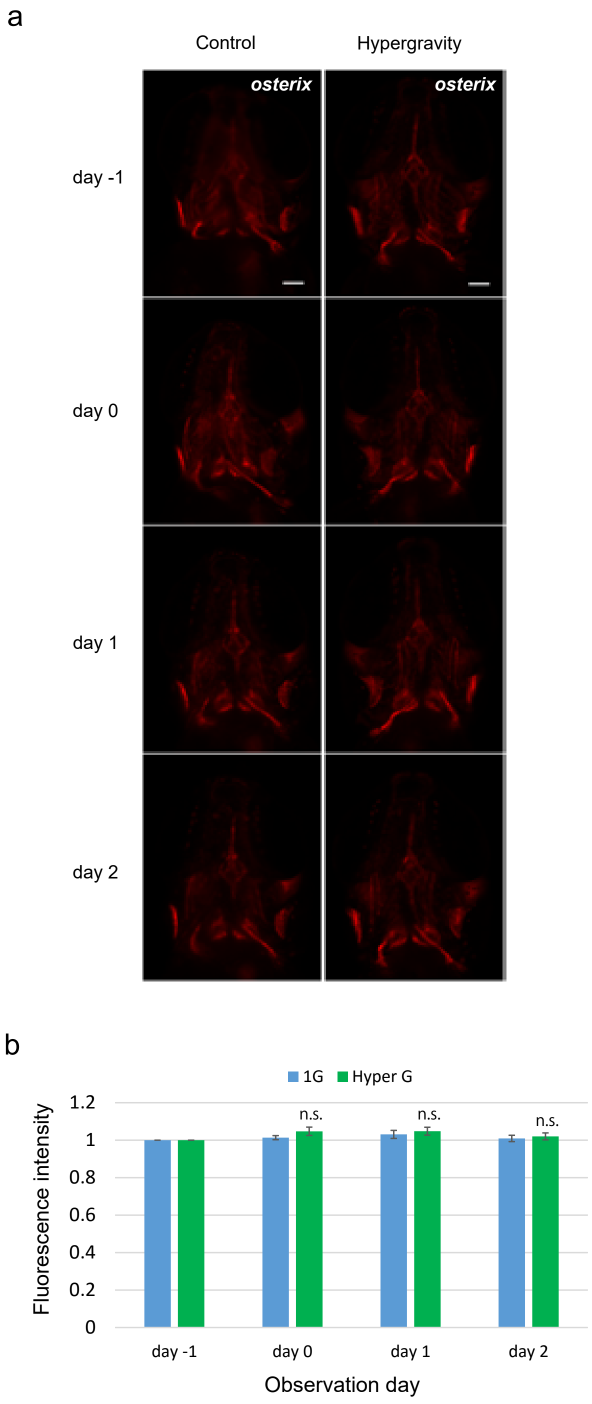

Supplementary Figure S2

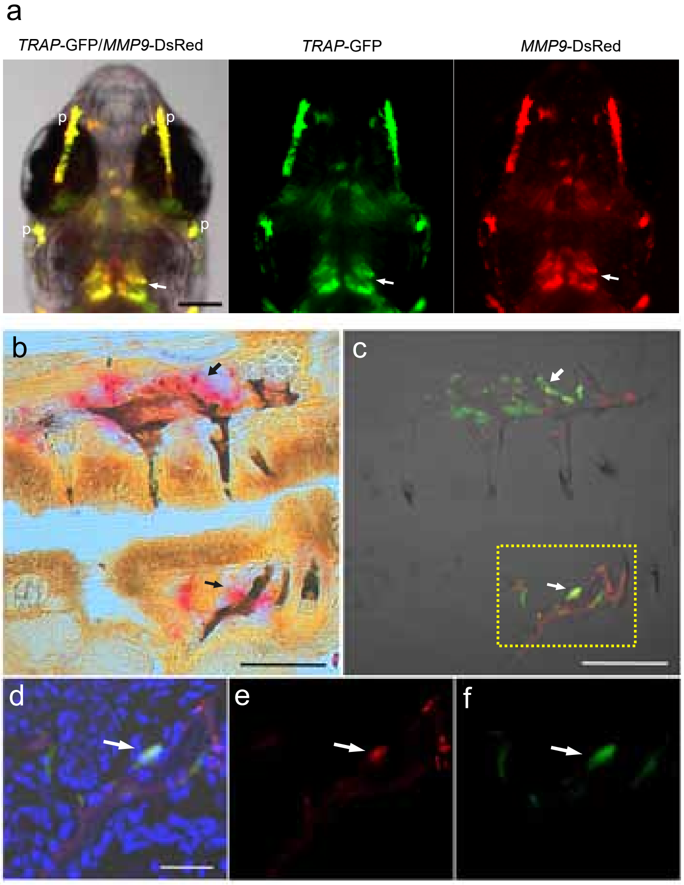

Supplementary Figure S3

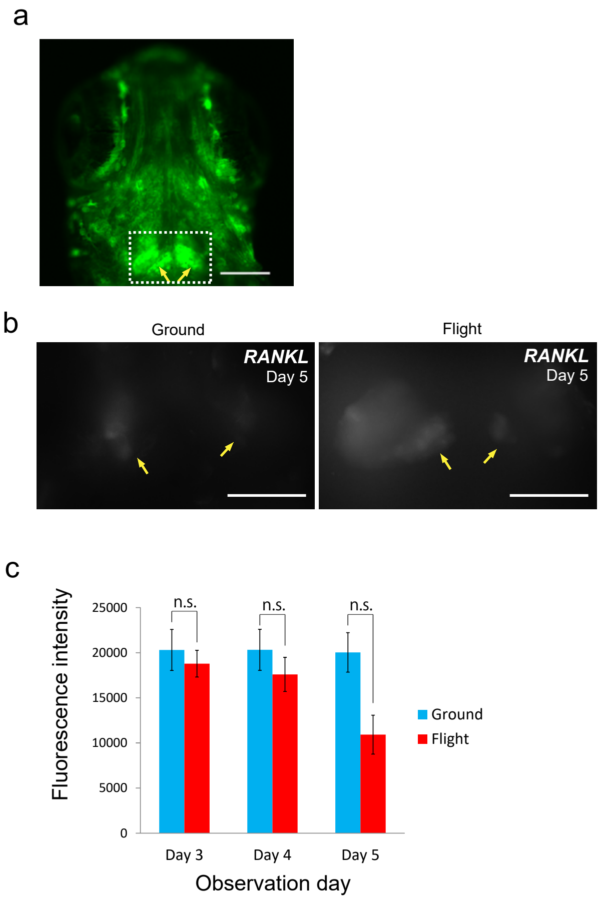

Supplementary Figure S4

Ground			Flight		
Day	N	Animal ID for *Osterix-DsRed*	Day	N	Animal ID for *Osterix-DsRed*
Day 1	9	A1, A2, A4, A6, A7, A8, A9, A10, A11	Day 1	6	a1, a2, a3, a4, a5, a6
Day 2	10	A1, A2, A4, A5, A6, A7, A8, A9, A10, A11	Day 2	6	a1, a2, a3, a4, a5, a6
Day 3	10	A1, A2, A4, A5, A6, A7, A8, A9, A10, A11	Day 3	6	a1, a2, a3, a4, a5, a6
Day 4	10	A1, A2, A4, A5, A6, A7, A8, A9, A10, A11	Day 4	7	a1, a2, a3, a4, a5, a6, a7
Day 5	11	A1, A2, A4, A5, A6, A7, A8, A9, A10, A11, A12	Day 5	7	a1, a2, a3, a4, a5, a6, a7
Day 6	10	A1, A4, A5, A6, A7, A8, A9, A11, A12, A13	Day 6	6	a1, a3, a4, a5, a7, a8
Day 7	11	A1, A4, A5, A7, A8, A9, A12, A14, A15, A16, A17	Day 7	5	a1, a3, a5, a7, a8
Day 8	11	A1, A4, A7, A9, A12, A14, A15, A16, A17, A18, A19	Day 8	4	a3, a5, a7, a8
Day	N	Animal ID for *Osteocalcin-DsRed*	Day	N	Animal ID for *Osteocalcin-DsRed*
Day 1	6	B1, B2, B3, B4, B5, B6	Day 1	3	b1, b2, b3
Day 2	7	B1, B2, B3, B4, B5, B6, B8	Day 2	3	b1, b2, b3
Day 3	7	B1, B2, B3, B4, B5, B6, B8	Day 3	5	b1, b2, b3, b4, b5
Day 4	6	B2, B4, B5, B6, B8, B10	Day 4	4	b2, b3, b4, b5
Day 5	3	B4, B8, B10	Day 5	4	b2, b3, b4, b5
Day	N	Animal ID for *TRAP-GFP*	Day	N	Animal ID for *TRAP-GFP*
Day 4	14	A1, A2, A3, A4, A6, A7, A8, A9, A10, A11, B4, B5, B8, B10	Day 4	9	a1, a2, a3, a4, a5, a6, a7, b2, b3
Day 6	16	A1, A2, A4, A6, A7, A8, A9, A11, A12, B4, B8, B10, B12, B13, B14, B15	Day 6	7	a1, a3, a4, a5, a7, a8, b3
Day	N	Animal ID for *MMP9-DsRed*	Day	N	Animal ID for *MMP9-DsRed*
Day 4	6	C1, C2, C3, C4, C5, C6	Day 4	4	c1, c2, c3, c4
Day 6	6	C1, C2, C3, C4, C5, C6	Day 6	3	c2, c3, c4
Day	N	Animal ID for *RANKL-GFP*	Day	N	Animal ID for *RANKL-GFP*
Day 3	6	C1, C2, C3, C4, C5, C6	Day 3	3	c1, c2, c3
Day 4	6	C1, C2, C3, C4, C5, C6	Day 4	3	c1, c3, c4
Day 5	6	C1, C2, C3, C4, C5, C6	Day 5	3	c1, c3, c4
Day	N	Animal ID for *Osterix-DsRed* (Whole-body)	Day	N	Animal ID for *Osterix-DsRed* (Whole-body)
Day 1	5	A1, A2, A3, A4, A5	Day 1	3	a1, a4, a8
Day 2	5	A1, A2, A3, A4, A5	Day 2	3	a1, a4, a8
Day 4	5	A1, A2, A3, A4, A5	Day 4	4	a1, a2, a4, a8
Day 6	5	A1, A2, A3, A4, A5	Day 6	3	a1, a2, a8
Day 7	5	A2, A3, A4, A6, A7	Day 7	3	a1, a2, a8

Supplementary Table S1 | The record of observation for individual medaka samples ($n \geqq 3$) in the ground control (left) and flight (right) group

"A-C" and "a-c" show the kind of transgenic lines for observation; Osterix-DsRed/TRAP-GFP transgenic line in the ground (A) and flight (a), Osteocalcin-DsRed/TRAP-GFP transgenic line in the ground (B) and flight (b), and MMP9-DsRed/RANKL-GFP transgenic line in the ground (C) and flight (c). "Day" means the observation day. "N" means the number of observed fish. The images of ground control samples were captured during 2014/06/09-2014/0616, and those of flight samples were captured during 2014/02/07-2014/02/14.

Figure number	Object	Ground		Flight		Objective lens
		Gain value	Exposure time (msec)	Gain value	Exposure time (msec)	
Figure 2c, d	Osterix-DsRed (Whole-body)	1.0	300	1.0	300	5x
Figure 3a, b	Osterix-DsRed	1.0	800	1.0	800	20x
Figure 3e, f	Osteocalcin-DsRed	1.0	3000	1.0	3000	20x
Figure 4a, b	TRAP-GFP	1.0	300	1.0	300	20x
Figure 4d, e	MMP9-DsRed	1.0	3000	1.0	3000	20x
Figure S4b	RANKL-GFP	1.0	300	1.0	300	20x

Supplementary Table S2 | Fluorescence observation for the flight and ground control samples
Both samples were observed under the same conditions.

Up-regulated GO annotations	q-value	Down-regulated GO annotations	q-value
Molecular function		*Molecular function*	
DNA binding	0.0175393	extracelullar matrix structural constituent	0.00401414
Heterocyclic compound binding	0.0157555	phosphotransferase activity, alcohol group as acceptor	0.00974622
Nucleic acid binding	0.0000912	protein kinase activity	0.0195225
Organic cyclic compound binding	0.0157555	receptor activity	0.00401414
Biological process		*Biological process*	
Cellular aromatic compound metabolic process	0.00896628	muscle tissue development	0.0205161
Cellular nitrogen compound metabolic process	0.00675267	phosphorylation	0.0293491
DNA metabolic process	0.00675267	signal transduction	0.0205161
DNA replication	0.00675267	striated muscle tissue development	0.0293491
DNA replication initiation	0.00146611	*Cellular component*	
Endoderm formation	0.043615	collagen trimer	0.00374939
hematopoietic or lymphoid organ development	0.0269695	contractile fiber part	0.0301749
heterocycle metabolic process	0.00675267	extracellular matrix	0.000699573
nitrogen compound metabolic process	0.00675267	integral component of membrane	0.00000000322
nucleic acid metabolic process	0.00675267	integrin complex	0.0360586
nucleobase-containing compound metabolic process	0.00912311	membrane	0.00000000322
organic cyclic compound metabolic process	0.00675267	membrane part	0.000000403
Cellular component		plasma membrane	0.0360586
MCM complex	0.0124825	protein complex involved in cell adhesion	0.0360586
nucleus	0.000302051	proteinaceous extracellular matrix	0.00210483
		receptor complex	0.0360586

Supplementary Table S3 | Enriched systems by GO analysis for whole-body in-flight medaka at days 2 and 6

Gene ID	Gene title	FC (Day 2, F/G)	FC (Day 6, F/G)	p-value (Day 2)	p-value (Day 6)
LOC101166722	Krueppel-like factor 9-like	11.84	1.84	0.00005	0.0061
LOC101166590	TNFAIP3-interacting protein 1-like	6.74	2.44	0.00005	0.0185
LOC101164399	GATA-binding factor 5-A-like	6.13	3.04	0.00875	0.00665
LOC101156885	G0/G1 switch protein 2-like	5.84	2.96	0.00005	0.00005
LOC101157884	cAMP-responsive element modulator-like	5.40	1.99	0.00005	0.0173
LOC101166121	tubulin beta-6 chain-like	5.38	1.82	0.00025	0.01305
LOC101175275	serine/threonine-protein kinase SIK2-like	4.80	1.75	0.00005	0.0147
LOC101169519	early growth response protein 1-B-like	4.53	2.01	0.0043	0.0442
LOC101162685	unconventional myosin-XV-like	4.45	2.37	0.0003	0.00145
LOC101173775	transcription factor Maf-like	4.44	1.79	0.0007	0.02895
LOC101170604	PR domain zinc finger protein 1-like	4.18	1.80	0.00075	0.0413
foxa3	Me-FKH1	4.04	1.73	0.00005	0.0284
gata-1	hematopietic transcription factor GATA-1	3.68	1.87	0.00005	0.0037
LOC101170127	Krueppel-like factor 2-like	3.62	1.63	0.00005	0.0392
LOC101155601	CCAAT/enhancer-binding protein beta-like	3.62	2.40	0.00005	0.0037
LOC101164062	transcription factor jun-B-like	3.58	2.52	0.00005	0.00315
LOC101168009	jun dimerization protein 2-like	3.44	2.03	0.00005	0.00085
LOC101172251	neuronal PAS domain-containing protein 4-like	3.33	2.45	0.00055	0.00435
LOC101174822	transcription factor AP-1-like	3.32	1.65	0.00005	0.0217
LOC101169722	early growth response protein 1-like	3.15	2.4	0.00005	0.00015
LOC101175263	nuclear receptor subfamily 0 group B member 2-like	3.09	3.03	0.00005	0.00005
c-fos	c-fos	3.08	2.22	0.00005	0.002
LOC101168635	dnaJ homolog subfamily B member 5-like	3.00	2.66	0.00005	0.00005
LOC101156434	nuclear receptor subfamily 1 group D member 2-like	2.93	1.68	0.00005	0.01375
LOC101156720	r3hdm1 R3H domain containing 1	2.85	2.02	0.00005	0.0018
LOC101172767	CCAAT/enhancer-binding protein delta-like	2.84	2.24	0.00005	0.00335
LOC101163664	C8orf4	2.67	2.18	0.00005	0.0016
LOC101161414	TSC22 domain family, member 3	2.41	1.80	0.00055	0.0426
LOC101162015	NF-kappa-B inhibitor alpha-like	2.35	2.28	0.00015	0.00045
LOC101163907	signal-transducing adaptor protein 1-like	2.27	1.80	0.00025	0.00585
LOC101159693	vesicle-associated membrane protein 2-like	2.14	1.98	0.00345	0.00285
LOC101165223	GADD45 beta-like	2.56	2.99	0.00005	0.0003
LOC101166663	zinc finger protein 143-like	2.25	2.46	0.0004	0.00065

Supplementary Table S4 | Up-regulation of nucleus-related genes in whole-body in flight (F) versus ground (G) at days 2 and 6

*FC means fold change

Gene ID	Gene title	FC (Day 6, F/G)	p-value	q-value
LOC101173930	mcm2	1.79	0.01155	0.0838772
LOC101165815	mcm7	2.04	0.00185	0.026006
LOC101167880	mcm5	2.69	0.00005	0.00194545
LOC101155053	mcmbp	2.17	0.0012	0.0192805
LOC101159855	mcm6	1.81	0.00985	0.0764144
LOC101173977	mcm4	2.46	0.0001	0.00340372
LOC101165859	mcm3	2.79	0.00005	0.00194545

Supplementary Table S5 | Up-regulation of MCM family in whole-body medaka at day 6 in flight (F) versus ground (G)

*FC means fold change

Gene ID	Gene title	FC Day 2	FC Day 6	p-value Day 2	p-value Day 6
	Cell Adhesion				
LOC101166566	claudin-5-like	3.27	2.48	0.00005	0.00005
LOC101156528	claudin-7-like	-2.46	-4.17	0.014	0.00005
LOC101164424	claudin-like protein ZF-A89-like	-4.20	-3.11	0.0073	0.00035
LOC101159003	desmoglein-4-like	-7.41	-17.15	0.00305	0.00005
LOC101165224	desmoplakin-like	-3.01	-8.22	0.00035	0.00005
LOC101160163	neural cell adhesion molecule L1-like protein-like	-2.38	-3.29	0.0176	0.00005
LOC101156181	carcinoembryonic antigen-related cell adhesion molecule 1-like	-3.68	-5.21	0.00255	0.00005
LOC101174027	integrin alpha-2-like	-3.51	-2.73	0.00415	0.00005
	Channel, Transporter				
LOC101160832	solute carrier family 13 member 5-like	15.56	2.03	0.00005	0.02465
LOC101164809	solute carrier family 25 member 38-A-like	3.25	2.06	0.00005	0.0011
LOC101161366	solute carrier family 12 member 3-like	-7.57	-3.23	0.00005	0.00005
LOC101163089	aquaporin-3-like	-1.75	-4.23	0.0183	0.00005
LOC101156245	ammonium transporter Rh type B-A-like	-3.36	-3.29	0.01405	0.00805
	Receptor				
LOC101155005	sphingosine 1-phosphate receptor 1-like	-2.71	-3.66	0.01175	0.00005
LOC101154894	macrophage mannose receptor 1-like	-2.45	-3.56	0.0192	0.00005
	Membrane-related				
LOC101166070	transmembrane protease serine 4-like	-10.85	-4.79	0.008	0.00005
LOC101170062	class I histocompatibility antigen, F10 alpha chain-like	-8.88	-8.22	0.0123	0.00005
LOC101173791	chondroitin sulfate proteoglycan 4-like	-2.99	-2.39	0.01025	0.00095
LOC101155676	regulator of G-protein signaling 1-like	-2.89	-2.69	0.008	0.00005
LOC101172542	cell cycle control protein 50B-like	-1.96	-1.96	0.00265	0.00195
LOC101156171	gastric intrinsic factor-like	-4.41	-14.72	0.00005	0.00005
LOC101163639	C-type lectin domain family 4 member M-like	-2.08	-3.39	0.00505	0.00005
LOC101165853	abca12 ATP-binding cassette, sub-family A (ABC1), member 12	-2.16	-3.18	0.01155	0.00005
LOC101173994	probable G-protein coupled receptor 116	-2.62	-2.83	0.0104	0.00005
LOC101158220	calpain-5-like	-2.19	-2.51	0.00585	0.00005
LOC101173105	kunitz-type protease inhibitor 1-like	-1.72	-2.17	0.01795	0.0014
LOC101159307	transmembrane protein 54-like	-1.79	-2	0.01855	0.00125

Supplementary Table S6 | Alteration of the expression of membrane-related genes in whole-body medaka at days 2 and 6 in flight (F) versus ground (G)

*FC means fold change
